# CT-based dentulous mandibular alveolar ridge measurements as predictors of crown-to-implant ratio for short and extra short dental implants

**DOI:** 10.1038/s41598-020-73180-3

**Published:** 2020-10-01

**Authors:** Stefano Sivolella, Silvia Meggiorin, Nadia Ferrarese, Amalia Lupi, Francesco Cavallin, Antonino Fiorino, Chiara Giraudo

**Affiliations:** 1 Department of Neurosciences, Dentistry Section, University of Padova, Via Giustiniani, 1, 35131 Padua, Italy; 2 Department of Medicine-DIMED, Institute of Radiology, University Hospital of Padova, Padua, Italy; 3 Cellular and Molecular Clinical Research, Dentistry Unit of Head and Neck Clinical Area, School of Dentistry, Catholic University of Sacred Heart, Rome, Italy; 4 Solagna, Italy

**Keywords:** Bone, Mandibular muscles

## Abstract

The purpose was to predict the crown-to-implant ratio variation in the edentulous posterior mandibles rehabilitated with short dental implants. Hence, vertical and horizontal dimensions of dentulous posterior mandibles in a sample of 18- to 25-year-olds were measured, and correlations of these dimensions with sex and site were investigated. Mandibular computed tomography scans from 100 subjects were considered. Vertical and horizontal bone and tooth measurements were taken at the sites of the second premolar (PM), and the mesial and distal roots of the first and second molars (M1m, M1d, M2m and M2d, respectively). A hypothetical crown-to-implant ratio (C/I R) was calculated assuming the insertion of short and extra short implants (5, 6 or 7 mm), at 1.5 mm from the inferior alveolar canal, maintaining the position of the existing occlusal plane. All vertical bone dimensions decreased from the PM to the M2d. Width measurements increased from the mesial (PM) to the distal sites (M1m, M1d, M2m and M2d). Males had significantly greater vertical and horizontal measurements than females at all sites. The mean C/I R was higher than 2 for all sizes of implant. The C/I R was lower for the second molar than for the second premolar, while it was similar for the first molar and the second premolar. Males had a higher C/I R than females. Computed tomography can be used to study the anatomical features of alveolar bone, and to predict some clinical aspects of prosthetic rehabilitation with implants, such as the crown-to-implant ratio in conditions of serious bone atrophy.

## Introduction

The position and morphology of the inferior alveolar canal, and the dimension of the alveolar bone have been studied both in two-dimensional radiography and three-dimensional computed tomography. It has proved useful for examining whether a correlation exists between these measurements and other variables, such as sex and age^1–7^. Previous descriptive studies investigated the role of CT imaging in providing preoperative information in the oral and implant surgery setting too^8,9^. The above-mentioned literature focused on both dentulous^1–4,8^ and edentulous subjects^3–9^. Analyzing the dimensions of the alveolar ridge in young dentulous patients helped to shed light on the bone remodeling process, to predict the need for any bone grafting, and to orient the choice of the most appropriate size of implant. Such dimensional assessments ultimately facilitated the prosthetic rehabilitation of edentulous patients.

A classification of the edentulous jaws has been developed by Cawood and Howell^10^ based on a randomized cross-sectional study from a sample of 300 dried skulls, with the aim to anticipate and avoid future clinical problems. It has been reported^11^ that the distance between the mandibular canal and the cranial edge of the body of the posterior edentulous mandible is between 12 mm (SD 3.7 mm) and 8.83 mm (SD 3.9 mm) for Cawood and Howell^10^ class V mandibular atrophy, and between 9.67 mm (SD 2.4 mm) and 2.61 mm (SD 1.6 mm) for class VI. Class IV, V and VI could require a bone graft to augment the alveolar ridge height followed by the placement of a "long" implant, or, in alternative, the use of "extra short" implants. As regards dental implants’ length, the following classification has been proposed by Al-Johany et al.^12^: considering the designed intra-bony length of a dental implant, the terms extra short (≤ 6 mm), short (> 6 mm to < 10 mm), standard (≥ 10 mm to < 13 mm) and long (≥ 13 mm) have been presented.

Standard length implants placed in vertically regenerated posterior mandibular sites has been compared to short and extra short implants in the severely resorbed mandible, in terms of implant and prosthetic survival, marginal bone resorption and morbidity^13–18^.

The common conclusion of these works is that when residual bone height over the mandibular canal is between 7 and 8 mm, short and extra short implants might be a preferable treatment option over vertical augmentation, reducing chair time, expense, and morbidity. Studies with four to five years follow up on implants with length between 4 and 6 mm report acceptable survival rate values between 86.7% and 97.8%^19–23^.

Various studies have shown the same conclusion for the maxilla as well. The comparison between standard length implants positioned after sinus augmentation with bone graft towards short implants led to an equal result in terms of implant survival, reducing biological complications, morbidity, costs and surgical time^24–26^.

This therapeutic option^27^ based on short and extra short dental implants, supporting fixed prosthetic rehabilitations, is associated with an increase in prosthetic crown height related to the implant length (crown-to-implant ratio, C/I R)^28–30^.

Two different versions of the C/I R have been described, depending on the apico-coronal placement of the fulcrum:^31^ the anatomical C/I R, where the fulcrum of the lever arm is located at the implant shoulder; and the clinical C/I R, where the fulcrum lies within the bone crest. In the latter case, the length of the crown may include the part of implant that may not be completely embedded in the bone.

Prosthetic rehabilitations on short implants are often associated with higher C/I Rs, which may exacerbate bone loss, or even cause failure of the implant^32–34^.

Data in the literature indicate that the maximal C/I R ranges from 3^35,36^ to 4.95^37^. No critical threshold has been established for the C/I R in order to avoid excessive bone loss or implant failure, and it has been variously proposed in the range of 1.46^38^ to 3.10^39^. On the other hand, many studies found no correlation between higher C/I Rs and higher rates of prosthetic complications, marginal bone loss, or implant failure^30,40–51^.

The present study hypothesis was as follows. We can measure the dimensions of a given individual’s posterior mandibular alveolar bone and tooth, and the position of the occlusal plane. Then we can imagine this individual having become edentulous and consequently presenting with severe bone resorption. If it is still feasible to insert a short or extra short implant (5, 6 or 7 mm long) at least 1.5 mm away from the mandibular canal, then we can calculate the corresponding C/I R. This hypothesis is supported by the fact that the occlusal vertical dimension remains constant throughout an individual’s life^52–55^.

The aim of the present observational study was to predict a C/I R for the hypothetical positioning of a short or extra short implant 1.5 mm away from the mandibular canal, and to see if any correlations exist between the C/I R, the implant site, and sex. Hence, the alveolar bone dimensions in the posterior mandible of a sample of dentulous 18- to 25-year-olds were measured with the aid of CT, and any correlation between these measurements and sex were investigated.

## Materials and methods

### Study design

Mandibular dental CT scans of 147 subjects referring to the Radiology Department of Padova University Hospital from 2008 to 2016 were anonymously analyzed for this retrospective study.

All datasets were acquired using a multislice (64 slices) CT scanner (Somatom Sensation, Siemens GmbH Healthcare, Erlangen, Germany).

The following inclusion criteria were applied: (1) age between 18 and 25 years; (2) a fully dentulous mandible; (3) no fillings or endodontic treatments involving the second premolar and/or the first and second molars. CT images were excluded if at least one of the following criteria was met: (1) complete osseous retention of one or more of the observed teeth in the mandible; (2) radiographic evidence of prior bone augmentation procedures or signs of invasive surgery; (3) presence of pathological radiolucent or radiopaque areas; (4) partial or complete osseous retention of one or more residual roots; (5) presence of osteosynthesis plaques; (6) dataset affected by artifacts (e.g., motion artifacts concealing the mandibular canal). Only one scan was selected and examined per subject.

Once the relevant exams have been identified, the related dicom files have been saved anonymously and analyzed with dedicated software. Access to the x-ray archive, patient selection and saving of dicom files were performed by a single operator (SS). No information has been linked to the dicom files, if not age and gender. No patient/code association register has been generated. No member of the research team named in the author list of the paper had access to identifying subjects while analyzing the data. This retrospective analysis of routine anonymized clinical data was approved by the local Ethic Committee (protocol n. 35725). The need for informed consent was waived by the local ethics committee. All procedures were in accordance with the ethical standards of the 1964 Helsinki Declaration and its later amendments or comparable ethical standards.

The STROBE guidelines for the compilation of this article have been followed.

### CT scan processing and radiographic measurements

**Figure 1:**
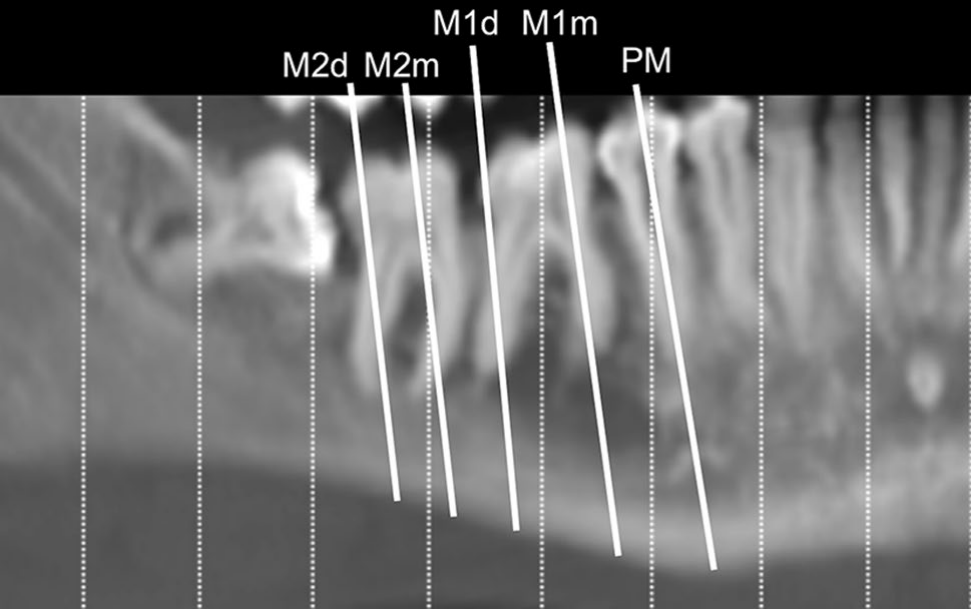
Schematic view on a panoramic image of the images selected on a level with the second premolar, and the mesial and distal roots of the first and second molars (sites PM, M1m, M1d, M2m and M2d, respectively).

The measurements of interest were taken on both sides of each dentulous mandible from one cross-sectional image obtained by CT multiplanar reformation (CT/MPR) on a level with each second premolar, and the mesial and distal roots of the first and second molars (these sites were named PM, M1m, M1d, M2m and M2d, respectively) (Fig. 1). Seven lines were traced parallel to the occlusal plane (o) on each image: line 1 touched the inferior border of the mandible (basal bone, b); line 2 was at the most coronal point of the alveolar crest (crest, c); and lines 3–7 were placed at a height of 1, 3, 5 and 10 mm apical to the c (h_1_, h_3,_ h_5,_ and h_10_, respectively). The most coronal point of the mandibular canal (mandibular canal, mc), and the most coronal point of the crown of the tooth considered (occlusal, o) were identified.

**Figure 2:**
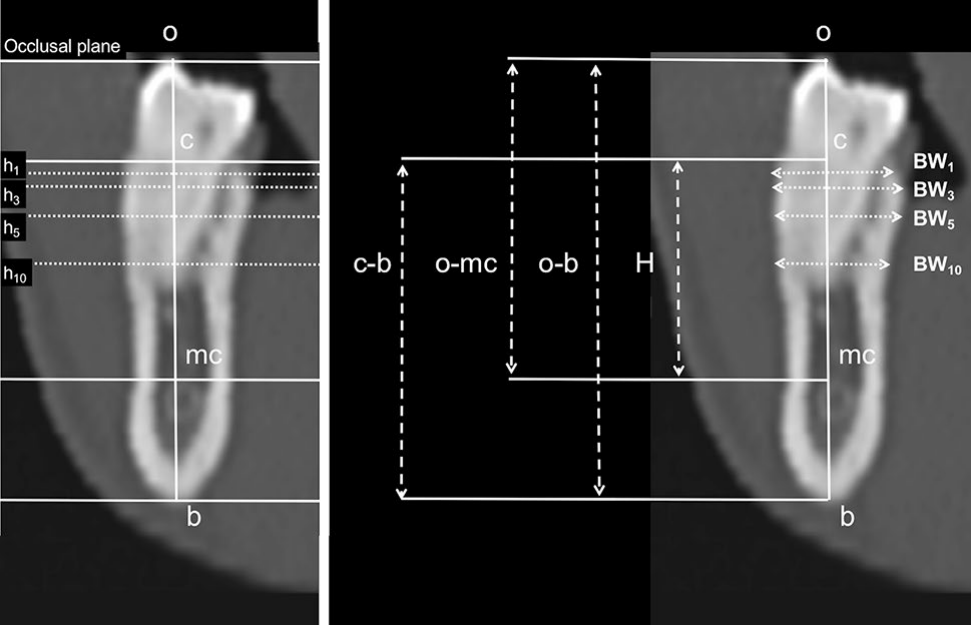
Seven lines parallel to the occlusal plane were traced on each cross-sectional image: line 1 touching the inferior border of the mandible (basal bone, b); line 2 at the most coronal point of the alveolar crest (crest, c); lines 3 to 6 placed at 1, 3, 5 and 10 mm apical to c (h_1_, h_3 ,_ h_5_ and h_10_, respectively); and line 7 at the most coronal point of the mandibular canal (mandibular canal, mc); and the most coronal point of the crown of the tooth considered (occlusal, o) was identified on the selected cross-sectional images.

On the selected images (PM, M1m, M1d, M2m, M2d), the following vertical measurements (bone heights, BHs) were taken along a line perpendicular to the occlusal plane (Fig. 2):

- the distance between the occlusal plane and the most coronal point of the mandibular canal (o-mc);
- the distance between the occlusal plane and the basal bone (o-b);
- distance between the crest and the basal bone (c-b);
- the distance (H) between the most coronal point of the alveolar crest (c) and the most coronal point of the mandibular canal (mc) was calculated by subtracting the values as follows: H(c-mc) = c-b– (o-b – o-mc).

Bucco-lingual bone widths (BWs) were measured at h_1_, h_3_, h_5_, and h_10_, (BW_1_, BW_3_, BW_5_ and BW_10_, respectively).

A single mean dimension was then calculated for each measurement on a level with M1m-M1d, and with M2m-M2d, indicated respectively as M1 and M2.

**Figure 3:**
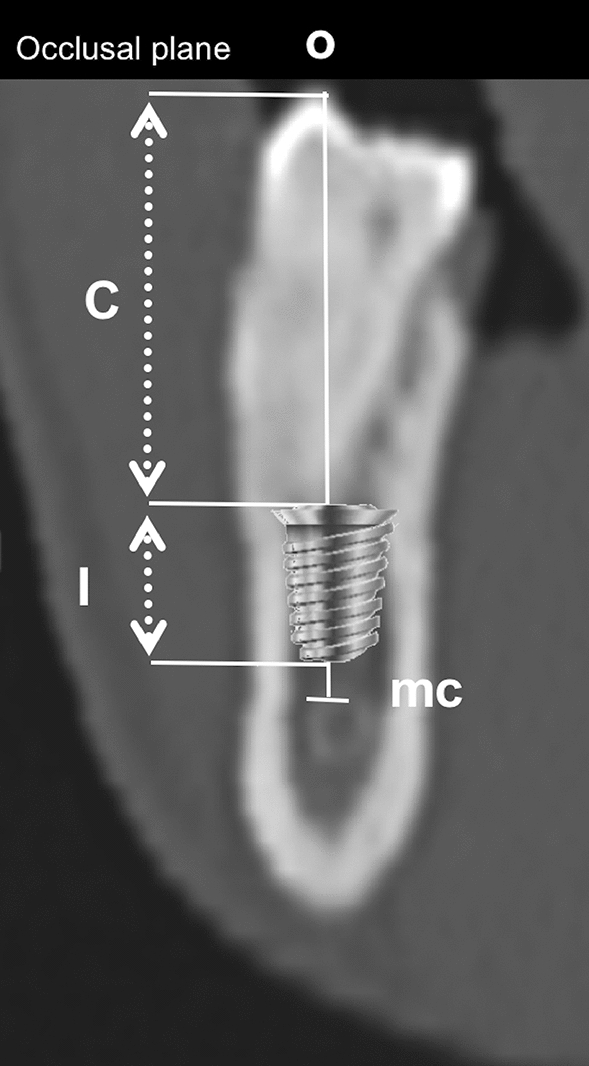
Hypothetical short dental implant placed 1.5 mm coronally to mc, and consequent clinical crown length calculation assuming the occlusal plane remains in the same position as in the dentulous condition.

Some additional calculations were completed, based on the findings of the present study. Assuming that the teeth considered (PM, M1, M2) had been lost, and a dental implant was to be inserted a safe distance (usually deemed to be 1.5 mm from the mandibular canal)^56^, we calculated the anatomical C/I R in conditions of vertical bone atrophy, maintaining the existing occlusal plane. This calculation was based on the insertion of a short or extra short dental implant 5, 6 or 7 mm long at the PM, M1 and M2 mandibular sites. Anatomical C/I R was calculated as the ratio between the dimension C (distance between the coronal top of the implant and the occlusal plane) and I (the length of the chosen implant—5 mm, 6 mm or 7 mm) (Fig. 3).

Then a single value for each BH, BW measurement and C/I R calculation was obtained for the PM, M1 and M2 on the right and left sides.

### Statistical analysis

Continuous data were expressed as means and standard deviations (SD), and categorical data as numbers and percentages. A multivariable analysis on the main outcomes (BWs, BHs and C/I Rs) was run using multilevel models to take into account the inclusion of multiple teeth per subject. One multilevel multivariable model was estimated to identify the BW predictors among the clinically-relevant variables (site, sex, and depth of measurement). Four multilevel multivariable models were estimated to identify the predictors of o-mc, o-b c-b, and H (c-mc) among the clinically-relevant variables (site and sex). One multilevel multivariable model was estimated to identify the predictors of the C/I R among the clinically-relevant variables (site, sex, and implant size). The initial models included all the single terms and interaction terms, and model selection was done by minimizing the Akaike information criterion (AIC). All tests were two-sided and a p-value below 0.05 was considered statistically significant. The statistical analysis was performed using R 3.3 (R Foundation for Statistical Computing, Vienna, Austria)^57^.

### Ethical approval

This retrospective analysis of routine anonymized clinical data was approved by the local Ethic Committee (Protocol n. 35725). The need for informed consent was waived by the local ethics committee. All procedures were in accordance with the ethical standards of the 1964 Helsinki Declaration and its later amendments or comparable ethical standards .

## Results

There were 147 CTs obtained during the selected time interval, but only 100 met the inclusion criteria and were examined for the purposes of this study. The sample of 100 subjects (median age 21 years, IQR 20–23) included 44 males (median age 21 years, IQR 19–22) and 56 females (median age 22 years, IQR 20–23). The measurements obtained are listed in Tables 1 and 2.

**Table 1:** Alveolar bone width (BW) measurements.

	PM				M1				M2			
	BW_1_	BW_3_	BW_5_	BW_10_	BW_1_	BW_3_	BW_5_	BW_10_	BW_1_	BW_3_	BW_5_	BW_10_
**Overall**												
Mean	9.04	9.97	10.33	10.74	10.69	11.72	12.16	12.42	11.90	13.44	14.12	14.39
SD	1.12	1.26	1.46	1.52	1.06	1.24	1.35	1.60	1.43	1.62	1.75	1.83
Max	13.11	14.45	15.89	16.17	15.84	16.83	16.58	17.16	17.40	18.20	19.00	19.48
Min	5.27	6.60	6.93	6.27	7.59	8.25	8.47	8.63	8.24	10.59	9.50	10.06
**Females**												
Mean	8.86	9.73	10.02	10.26	10.39	11.31	11.75	11.88	11.47	13.04	13.70	13.78
SD	1.19	1.33	1.41	1.27	0.87	1.04	1.06	1.14	1.17	1.42	1.53	1.47
Max	13.11	14.45	13.91	12.76	12.55	13.89	14.18	14.19	15.33	16.83	17.49	17.17
Min	5.28	6.60	6.93	6.27	7.59	8.25	8.47	8.63	9.23	10.70	10.45	10.48
**Males**												
Mean	9.28	10.28	10.72	11.35	11.08	12.25	12.69	13.09	12.45	13.93	14.66	15.16
SD	0.99	1.10	1.44	1.60	1.16	1.27	1.49	1.84	1.55	1.73	1.87	1.96
Max	11.56	13.05	15.89	16.17	15.84	16.83	16.58	17.16	17.40	18.20	19.00	19.48
Min	5.27	8.23	7.18	7.54	8.44	10.02	10.18	8.91	8.24	10.59	9.50	10.06

PM: second premolar; M1: first molar; M2: second molar; BW1, BW3, BW5 and BW10: measurements taken at h 1 mm, h 3 mm, h 5 mm and h 10 mm.

**Table 2:** Alveolar bone height (BH) measurements.

	PM				M1				M2			
	o-mc	o-b	c-b	H (c-mc)	o-mc	o-b	c-b	H (c-mc)	o-mc	o-b	c-b	H (c-mc)
**Overall**												
Mean	26.57	38.75	30.39	18.21	26.28	37.12	29.03	18.19	24.67	35.33	27.89	17.24
SD	2.40	3.08	2.79	2.28	2.21	3.03	2.93	2.32	2.60	3.27	2.98	2.57
Max	34.04	47.01	38.37	24.42	33.01	46.97	37.39	26.63	34.02	47.25	36.67	32.16
Min	20.74	31.67	22.31	10.28	19.58	29.11	15.61	4.07	15.68	26.64	19.24	10.70
**Females**												
Mean	25.79	37.20	29.14	17.73	25.68	35.83	27.82	17.68	24.11	34.19	26.79	16.72
SD	2.32	2.30	2.27	2.29	1.99	2.61	2.57	2.19	2.28	2.93	2.64	2.15
Max	31.40	42.28	34.19	23.07	30.95	41.81	33.94	26.63	32.76	43.68	33.67	22.69
Min	20.74	31.67	22.31	10.28	20.84	29.11	15.61	4.07	18.92	26.64	19.24	12.00
**Males**												
Mean	27.56	40.72	31.98	18.81	27.05	38.77	30.57	18.84	25.39	36.78	29.30	17.92
SD	2.12	2.82	2.60	2.14	2.25	2.73	2.63	2.34	2.80	3.11	2.81	2.88
Max	34.04	47.01	38.37	24.42	33.01	46.97	37.39	25.10	34.02	47.25	46.67	32.16
Min	22.76	34.52	26.28	13.80	19.58	31.29	23.71	23.71	15.68	26.65	21.44	10.70

PM: second premolar; M1: first molar ; M2: second molar; o-mc: distance between the occlusal plane and the most coronal point of the mandibular canal; o-b: distance between the occlusal plane and the basal bone; c-b: distance between the crest and the basal bone; H (c-mc) distance between the crest and the mandibular canal.

### Multilevel multivariable analysis of BW

Model selection in the multilevel multivariable analysis of the BW was performed by minimizing the AIC: the 3-way interaction term was removed (the AIC dropped from 7700 to 7670). In the final model (Table 3), BW was associated with site (*p* < 0.0001) and depth of measurement (*p* < 0.0001). The first and second molars were associated with an increase in mean BW of 1.62 and 2.93 mm with respect to the second premolar. The depth of measurement was associated with an increase in mean BW of 0.14 mm per mm of depth. The interaction terms site* depth of measurement (*p* < 0.0001), site*sex (*p* = 0.004) and sex* depth of measurement (*p* < 0.0001) were also associated with statistically significant increments in BW.

**Table 3:** Multivariable analysis of alveolar bone width (BW) measurements.

	*p* value	Regression coefficient (95% CI)
Intercept	< 0.0001	9.03 (8.74 to 9.32)
**Site**	< 0.0001	
PM		Reference
M1		1.62 (1.40 to 1.83)
M2		2.93 (2.72 to 3.15)
Depth of measurement	< 0.0001	0.14 (0.12 to 0.17)
**Sex**	0.05	
Female		Reference
Male		0.41 (0.00 to 0.81)
Site*depth of measurement	< 0.0001	
PM* depth of measurement		Reference
M1* depth of measurement		0.00 (− 0.03 to 0.03)
M2* depth of measurement		0.07 (0.4 to 0.11)
Site*sex	0.004	
PM*female		Reference
M1*male		0.25 (0.03 to 0.48)
M2*male		0.36 (0.14 to 0.58)
Sex*depth of measurement	< 0.0001	0.06 (0.03 to 0.08)

PM: second premolar. M1: first molar. M2: second molar. Model selection was done by minimizing the Akaike information criterion (AIC): the 3-way interaction term was removed (AIC from 7700 to 7670).

### Multilevel multivariable analysis of BH

Model selection in multilevel multivariable analysis of BH was performed by minimizing the AIC. Full models with the interaction term were estimated because removing the interaction term did not reduce the AIC. In the final models (Table 4), site (*p* < 0.0001) and sex (*p* < 0.01) were associated with all BH measurements. Males had a longer o-mc than females (mean difference 1.76 mm), while the second molar was associated with a decrease in o-mc with respect to the second premolar. Males had a longer c-b than females (mean difference 2.84 mm), while the first and second molars were associated with a decrease in c-b with respect to the second premolar. Males had a longer o-b than females (mean difference 3.52 mm), while the second molar was associated with a decrease in o-b with respect to the second premolar. In addition, the interaction term site*sex (*p* = 0.0004) showed a weaker effect of sex on o-b, depending on the site. Males had a longer H than females (mean difference 1.08 mm), while the second molar was associated with a decrease in H with respect to the second premolar.

**Table 4:** Multivariable analysis of alveolar bone height (BH) measurements.

	o-mc		o-b		c-b		H (c-mc)	
	p-value	Regression coefficient (95% CI)	p-value	Regression coefficient (95% CI)	p-value	Regression coefficient (95% CI)	p-value	Regression coefficient (95% CI)
Intercept	< 0.0001	25.79 (25.27 to 26.31)	< 0.0001	37.20 (36.51 to 37.88)	< 0.0001	29.14 (28.50 to 29.78)	< 0.0001	17.73 (17.22 to 18.25)
**Site**	< 0.0001		< 0.0001		< 0.0001		< 0.0001	
PM		Reference		Reference		Reference		Reference
M1		− 0.11 (− 0.49 to 0.27)		− 1.37 (− 1.68 to 1.07)		− 1.32 (− 1.62 to − 1.02)		− 0.06 (− 0.47 to 0.36)
M2		− 0.68 (− 2.06 to − 1.31)		− 3.00 (− 3.32 to − 2.70)		− 2.35 (− 2.65 to − 2.05)		− 1.03 (− 1.44 to − 0.61)
**Sex**	< 0.0001		< 0.0001		< 0.0001		0.007	
Female		Reference		Reference		Reference		Reference
Male		1.76 (0.98 to 2.55)		3.52 (2.49 to 4.56)		2.84 (1.87 to 3.81)		1.08 (0.30 to 1.86)
Site*sex	0.24		0.0004		0.28		0.92	
PM*female		Reference		Reference		Reference		Reference
M1*male		− 0.40 (− 0.97 to 0.17)		− 0.58 (− 1.05 to − 0.12)		− 0.10 (− 0.55 to 0.35)		0.09 (− 0.54 to 0.71)
M2*male		− 0.48 (− 1.05 to 0.09)		− 0.94 (− 1.40 to − 0.48)		− 0.33 (− 0.78 to 0.12)		0.13 (− 0.49 to 0.76)

PM: second premolar. M1: first molar. M2: second molar. o-mc: distance between the occlusal plane and the most coronal point of the mandibular canal o-b: distance between the occlusal plane and the basal bone. c-b: distance between the crest and the basal bone; H (c-mc) distance between the crest and the mandibular canal. Model selection was done by minimizing the AIC: full models with the interaction term were estimated because removing the interaction term did not reduce the AIC.

### Crown/implant ratio (C/I R)

The C/I R was calculated for three different hypothetical implant sizes (5, 6, and 7 mm). Overall, the mean C/I R was higher than 2 for all sizes of implant. The C/I Rs, by implant size, site and sex, are shown in Table 5.

**Table 5:** Calculation of the crown-to-implant ratio (C/I R), by implant length. PM: second premolar. M1: first molar. M2: second molar.

Implant size	Overall			PM			M1			M2		
	5 mm	6 mm	7 mm	5 mm	6 mm	7 mm	5 mm	6 mm	7 mm	5 mm	6 mm	7 mm
**Overall**												
Mean	3.88	3.07	2.49	4.01	3.18	2.58	3.99	3.16	2.66	3.65	2.87	2.32
SD	0.50	0.42	0.36	0.48	0.40	0.34	0.44	0.37	0.31	0.50	0.42	0.36
Max	5.51	4.42	3.65	5.51	4.42	3.65	5.25	4.21	3.47	4.86	3.88	3.19
Min	2.30	1.75	1.36	2.85	2.21	1.75	2.62	2.01	1.58	2.30	1.75	1.36
Proportion of CIR > 2	100%	99%	92%	100%	100%	96%	100%	100%	97%	100%	99%	84%
**Females**												
Mean	3.76	2.96	2.40	3.86	3.05	2.47	3.89	3.06	2.48	3.54	2.78	2.24
SD	0.46	0.38	0.33	0.46	0.39	0.33	0.38	0.32	0.27	0.45	0.38	0.32
Max	4.98	3.98	3.27	4.98	3.98	3.27	4.89	3.91	3.21	4.56	3.63	2.98
Min	2.60	1.99	1.57	2.85	2.21	1.75	2.87	2.22	1.76	2.60	2.00	1.57
Proportion of CIR > 2	100%	99%	89%	100%	100%	93%	100%	100%	96%	100%	99%	79%
**Males**												
Mean	3.76	3.21	2.60	4.21	3.34	2.72	4.14	3.28	2.67	2.79	2.99	2.42
SD	0.51	0.42	0.36	0.42	0.35	0.30	0.47	0.39	0.33	0.52	0.43	0.37
Max	5.51	4.42	3.65	5.51	4.42	3.65	5.25	4.21	3.47	4.86	3.88	3.19
Min	2.30	1.75	1.36	3.25	2.54	2.04	2.62	2.01	1.58	2.30	1.75	1.36
Proportion of CIR > 2	100%	99%	95%	100%	100%	100%	100%	100%	98%	100%	98%	89%

A multilevel multivariable regression model was estimated to identify the factors associated with the C/I R, among sex, site and implant, including all interactions. Model selection was performed by minimizing the AIC: the 3-way interaction term was removed (reducing the AIC from 704 to 689). In the final model (Table 6), C/I R was associated with site (*p* < 0.0001), sex (*p* < 0.0001) and implant size (*p* < 0.0001). The C/I R was lower for the second molar than for the second premolar (mean difference − 0.33), while it did not differ between the first molar and the second premolar. Males had a higher C/I R than females (mean difference 0.34), but this difference varied by site and implant size (site*sex *p* = 0.008, and implant*sex *p* = 0.03). Longer implants were associated with shorter C/I Rs, and this effect was mitigated in the second molar (implant*site *p* = 0.04).

**Table 6:** Multilevel multivariable regression model of the C/I R estimated to identify the factors associated with the C/I R (sex, site or implant length), including all interactions.

	*p* value	Regression coefficient (95% CI)
Intercept	< 0.0001	3.86 (3.78 to 3.95)
**Site**	< 0.0001	
PM		Reference
M1		0.02 (− 0.05 to 0.07)
M2		− 0.33 (− 0.39 to − 0.27)
**Sex**	< 0.0001	
Female		Reference
Male		0.34 (0.22 to 0.47)
**Implant size**	< 0.0001	
5 mm		Reference
6 mm		− 0.81 (− 0.87 to − 0.76)
7 mm		− 1.39 (− 1.45 to − 1.34)
Site*sex	0.008	
PM*female		Reference
M1*male		− 0.08 (− 0.14 to − 0.02)
M2*male		− 0.09 (− 0.15 to − 0.03)
Site*implant	0.04	
PM*5 mm		Reference
M1*6 mm		0.00 (− 0.07 to 0.07)
M2*6 mm		0.06 (− 0.01 to 0.13)
M1*7 mm		0.00 (− 0.07 to 0.07)
M2*7 mm		0.10 (0.03 to 0.18)
Implant*sex	0.03	
5 mm*female		Reference
6 mm*male		− 0.05 (− 0.11 to 0.01)
7 mm*male		− 0.08 (− 0.14 to − 0.02)

PM: second premolar. M1: first molar. M2: second molar. Model selection was done by minimizing the AIC: the 3-way interaction term was removed (AIC from 704 to 689).

## Discussion

In this observational study, CT images were used to measure ridge dimensions in the posterior mandible of dentulous 18- to 25-year-olds. Larger BW dimensions were associated with depth of measurement, males and molars (compared to second premolar), while lower BH dimensions were associated with females and molars (compared to second premolar).

Many studies have reported on the bone height and width of posterior sextants of the mandible, though their findings are difficult to compare because no standardized measurement method was used.

Several authors measured bone width on different levels, from the dental crest to the edge of the alveolar canal (Table 7), and concurred that it increased gradually from the premolar through to the third molar region of the mandible, in both dentulous and edentulous mandibles^2,6–9^. Studies in edentulous subjects^6,7^ found that BW was not influenced by sex or age. It is interesting to note that BW measurements taken at various heights in edentulous subjects were always compatible with the insertion of a standard-diameter dental implant, validating our method for calculating the C/I R. The BW in the edentulous is reportedly approximately half that of dentulous subjects, confirming the fundamental role of tooth loss in severe bone resorption.

**Table 7:** Comparison of bone width (BW) values in the literature.

Authors	Year	Type of study	Sample size	Dentulous or edentulous	site PM					Site M1m-M1d					Site M2m-M2d				
					CREST	BW1	BW3	BW5	BW10	CREST	BW1	BW3	BW5	BW10	CREST	BW1	BW3	BW5	BW10
Watanabe et al.^8^	2010	CT	79	Dentulous and edentulous				12.8 ± 2.9					13.9 ± 3.2					15.2 ± 2.8	
Alrahaimi et al.^6^	2015	CBCT	120	Single missing tooth			6.22 ± 1.96					6.51 ± 1.75					7.60 ± 2.08		
Braut et al.^2^	2012	CBCT	56	Dentulous	7.63					10.44–10.24					9.84–10.17				
Zhang et al.^9^	2015	CBCT	59	Dentulous and edentulous			10.9 ± 2.6	11.6 ± 2.2	10.9 ± 2.1			11.6 ± 2.5	12.8 ± 2.5	11.5 ± 2.4			12.3 ± 3.3	13.5 ± 2.1	11.5 ± 1.9
Bressan et al.^7^	2017	CBCT	136	Edentulous		3.80 ± 1.42	7.26 ± 1.98	9.21 ± 2.04			4.54 ± 1.74	8.27 ± 2.30	10.26 ± 2.08			5.39 ± 2.21	9.50 ± 2.44	11.39 ± 2.16	
Present study	2018	CT	100	Dentulous		9.04 ± 1.12	9.97 ± 1.26	10.33 ± 1.46	10.74 ± 1.06		10.69 ± 1.06	11.72 ± 1.24	12.16 ± 1.35	12.42 ± 1.60		11.90 ± 1.43	13.44 ± 1.62	14.12 ± 1.75	14.39 ± 1.83

Our data showed that BH dimensions were higher in males than in females, while BH dimensions decreased from premolar to molars. Similarly, a significant difference in bone height between males and females has been reported in the dentulous^1,2^, the edentulous^7^, and both types of individual (Table 8)^3–5,8,9^. On the other hand, Alrahaimi et al. found no significant difference between males and females regarding distances from the alveolar crest to the superior position of the mandibular canal in a study on jaws with single missing teeth^6^. The measurement most commonly taken in the literature is the distance between c and mc (H). With the exception of Levine et al.^1^, studies on dentulous subjects generated data similar to those presented here, even if our H values seem to be slightly higher. Yashar et al. reported marked differences in their H values at the first molar due to the highly variable states of dentition and ages of their study population (from 22 to 83 years old)^4^. This may confuse the issue when it comes to comparing data, so it would be best to choose studies with a well-defined population, as regards age and state of dentition.

**Table 8:** Comparison of bone height (BH) values in the literature.

Authors	Year	Type of study	Sample size	Dentulous or edentulous	Crest to inferior border (CB)			Crest TO MC (H)		
					PM	M1	M2	PM	M1	M2
Watanabe et al. ^8^	2010	CT	79	Dentulous and edentulous	28.9 ± 3.6	28.2 ± 3.5	27.6 ± 3.8	15.3–17.4		
De Oliveira Junior et al.^3^	2011	CT	50	Dentulous and edentulous	27.80 ± 2.64	27.10 ± 3.28	27.20 ± 3.83	16.10 ± 2.52	16.40 ± 2.65	16.70 ± 3.41
Zhang et al.^9^	2015	CBCT	59	Dentulous and edentulous	27.5 ± 3.2	24.6 ± 2.9	23.8 ± 3.3	16.5 ± 3.0	14.8 ± 2.4	12.8 ± 2.9
Levine et al.^1^	2007	CT	50	Dentulous (1st molar)					17.4 ± 3.3	
Frei et al.^5^	2004	Linear tomography	35	Partially edentulous					14.87 ± 3.3	
Alrahaimi et al.^6^	2015	CBCT	120	Single missing tooth				15.19 ± 2.12	14.53 ± 2.34	14.21 ± 2.23
Braut et al.^2^	2012	CBCT	56	Dentulous				13.49–14.82		
Yashar et al.^4^	2012	CT	195	Dentulous and edentulous				9.91–16.03		
Bressan et al.^7^	2017	CBCT	136	Edentulous				11.20 ± 4.03	10.70 ± 3.74	10.28 ± 3.33
Present study	2018	CT	100	Dentulous	30.39 ± 2.79	29.03 ± 2.93	27.89 ± 2.98	18.21 ± 2.28	18.19 ± 2.32	17.24 ± 2.57

To calculate the C/I R for three different hypothetical implant sizes (5, 6, 7 mm long), we assumed that the occlusal plane remained the same distance from the mandibular canal in the dentulous and edentulous subjects. In fact, the positional relationship of the mandible to the head is reportedly unaffected by the presence or absence of teeth, and it alone determines the height of the face^53^. Thompson and Brodie reported that the vertical dimension of the occlusion is constant and does not vary as we grow older^52^. Levartovsky et al. also wrote that dental wear has no influence on the vertical dimension of occlusion or the height of the face in modern human skulls^54^.

In the present study, the mean anatomical C/I R calculated was always higher than 2: it ranged from 2.24 (females, 7 mm implant, M2) to 4.21 (males, 5 mm implant, PM). Males had a higher C/I R than females, but this difference varied for different sites and implant sizes. The C/I R was lower for second molars than for second premolars, and similar between first molars and second premolars. Longer implants were naturally associated with lower C/I Rs, and this effect was mitigated for second molars.

The direct correlation between higher anatomical C/I Rs and the risk of biological and mechanical complications is still controversial, although most of the literature, particularly on short and extra short dental implants, suggests that this factor has little or no influence^29–31,40–51,58–62^.

Schulte et al. reported that the mean C/I R for 889 plateau-design single tooth implants was 1.3 (the maximum C/I R was 3), with an average survival rate of 98.2% over 2.3 years^36^. Rokni et al. found that most implants had a C/I R between 1 and 2, with an average of 1.5 (and a maximum C/I R of 3)^35^. The C/I R appeared to have no significant effect on crestal bone levels, suggesting that a C/I R of 1.5 or more is not detrimental to the health of the implant. In one study, the C/I R was found unassociated with peri-implant marginal bone loss in the premolar and molar region, but longer anatomical crowns were associated with more technical complications^63^. On the other hand, prosthetic rehabilitations on short implants were associated in some studies with a higher C/I R, which might be associated with a greater bone loss or implant failure^32–34^.

Malchiodi et al. claimed that the critical threshold for the anatomical C/I R to avoid stress at the bone-implant interface being capable of causing excessive bone resorption or implant failure was 3.10, while the threshold for the clinical C/I R was 3.40^39^. Hingsammer et al. recommended not exceeding 1.7 to avoid exacerbating early marginal bone loss^64^. Quaranta et al. considered a C/I R ≥ 1.46 a potential risk factor for single crown and abutment loosening, and a C/I R ≥ 2.01 a risk factor for abutment fractures in posterior regions^38^.

In a study on 81 subjects treated with 326 implants (58.6% of them 6–8 mm long) with a mean follow-up of 70.7 months, an increase in C/I R reportedly did not lead to a higher risk of crestal bone loss, or more implant or crown failures after the insertion of single-tooth locking-taper implant restorations^37^. The authors stated that, when the length of the crown is up to 4.95 times the length of the implant within bone, implants can be successfully restored as single-tooth replacements. They presented a table with implant and crown failures, and two of the 6 implant failures had a C/I R of 4.56 and 4.95—values are among the highest reported in the literature.

Overall, very heterogeneous samples were included in the studies considered, as regards implant site and prosthetic design of the suprastructure, making it difficult to draw any reliable conclusions. That said, if we compare the results of the present study with the values reported in the literature, we can say that the former is realistic and applicable from the anatomical standpoint. Another point to make is that the C/I R was lower for the second molar than for M1 or PM, and that males always had a higher C/I R than females. In other words, if we accept that a high C/I R negatively affects the biological and mechanical outcomes of an implant-prosthetic rehabilitation, then the second molar site and female sex could be seen as protective prognostic factors.

The study has some limitation that should be considered. First, the underlying assumption of severe atrophy may be considered an extreme assumption. In our hypothesis, this represents a worst-case scenario where placing short or extra short implants is inevitable, thus our findings suggest “lower-bound” implications in planning implant prosthetics. Second, only one researcher (SM) performed all measurements and repeatability assessment was not conducted. Third, included patients were highly selected in terms of age and ethnicity, so our results should be considered with caution and applied appropriately. On the other hand, other studies have shown that one of the most important factors influencing alveolar bone dimensions is tooth loss, rather than age^4,9^.

A final consideration regards the use of data coming from a MSCT rather than a CBCT. CBCT image quality is comparable or even superior to MSCT, with low radiation dose and high-resolution imaging^65^. In this study, no specific choice of method was made, but available resources were used.

## Conclusion

The results of this anatomical study appear consistent with other reports in the literature and allow for some predictions for the purposes of planning implant prosthetics, providing useful information for managing patients with various degrees of bone atrophy.

## Data availability statement

The datasets generated and analysed during the current study are available from the corresponding author on reasonable request.
