# Publisher Correction: CT-based dentulous mandibular alveolar ridge measurements as predictors of crown-to-implant ratio for short and extra short dental implants

**DOI:** 10.1038/s41598-021-86889-6

**Published:** 2021-03-26

**Authors:** Stefano Sivolella, Silvia Meggiorin, Nadia Ferrarese, Amalia Lupi, Francesco Cavallin, Antonino Fiorino, Chiara Giraudo

**Affiliations:** 1 Department of Neurosciences, Dentistry Section, University of Padova, Via Giustiniani, 1, 35131 Padua, Italy; 2 Department of Medicine-DIMED, Institute of Radiology, University Hospital of Padova, Padua, Italy; 3 Cellular and Molecular Clinical Research, Dentistry Unit of Head and Neck Clinical Area, School of Dentistry, Catholic University of Sacred Heart, Rome, Italy; 4 Solagna, Italy

Correction to: *Scientific Reports* 10.1038/s41598-020-73180-3, published online 01 October 2020

In the original version of this Article, the affiliations given for Francesco Cavallin were incomplete.

The correct affiliations are listed below:

Solagna, Italy

Francesco Cavallin is an independent statistician.

This error has now been corrected in the PDF and HTML versions of the Article.

